# Kyste vestigial retrorectal: a propos d'un cas

**DOI:** 10.11604/pamj.2015.22.387.8255

**Published:** 2015-12-29

**Authors:** Hedfi Mohamed, Jomni Taieb, Abdelhedi Cherif, Sassi Karim, Chouchene Adnene

**Affiliations:** 1 Service de Chirurgie Générale, Hopital des FSI La Marsa Tunisie

**Keywords:** IRM, chirurgie, kyste vestigial, MRI, surgery, vestigial cyst

## Abstract

Les tumeurs rétro rectales kystiques et solides sont très variées et rare chez l'adulte. Elles sont dominées par les chordomes qui sont des tumeurs à malignité locale essentiellement observées chez les sujets de sexe masculin, alors les tumeurs kystiques vestigiales, sont souvent bénignes et prédominante chez la femme. Ces kystes congénitaux sont souvent asymptomatiques. L'apparition de symptôme à type des douleurs ou des troubles neurologiques devrait faire suspecter une dégénérescence. Une éventuelle surinfection tumorale peut poser le problème de diagnostic différentiel avec un abcès fistuleux, de traitement différent. Nous rapportons une observation insolite d'une patiente âgée de 52 ans explorée pour une lésion kystique retro rectale kystique en rapport avec un kyste vestigial traité chirurgicalement par voie trans sacrée. On se propose à travers cette observation insolite d’étudier les aspects diagnostique, évolutif et thérapeutique de ces lésions rares.

## Introduction

Les tumeurs retro rectales constituent une entité rare, souvent bénignes, asymptomatiques, avec une prédilection féminine. Les kystes et les tumeurs d'origine vestigiale y sont les moins rares. Le diagnostic positif repose actuellement sur l'apport des moyens modernes d'imagerie. Le traitement est essentiellement chirurgical alors que leur pronostic est habituellement favorable.

## Patient et observation

**Figure 1:**
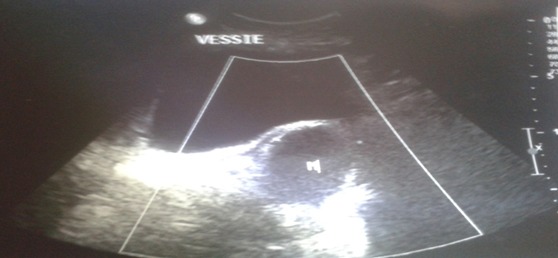
Echographie pelvienne montrant une formation kystique retro rectale de 6cm de diameter

**Figure 2:**
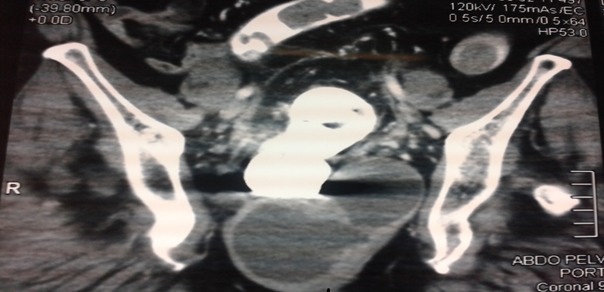
Coupe coronale d'un scanner pelvien montrant une masse hypodence retro rectal

**Figure 3:**
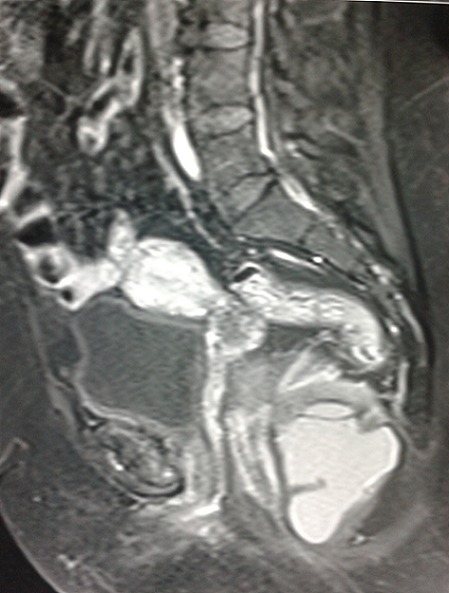
Coupe sagittale d'une IRM pelvienne montrant une lésion retro rectale hypo intense

**Figure 4:**
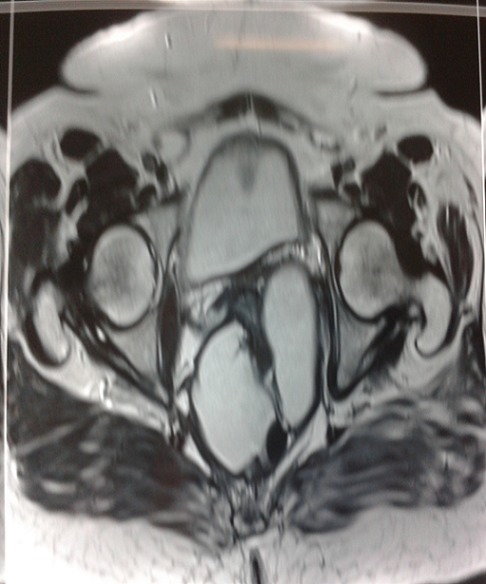
IRM pelvienne en pondération T1 montrant une lésion retro rectal

**Figure 5:**
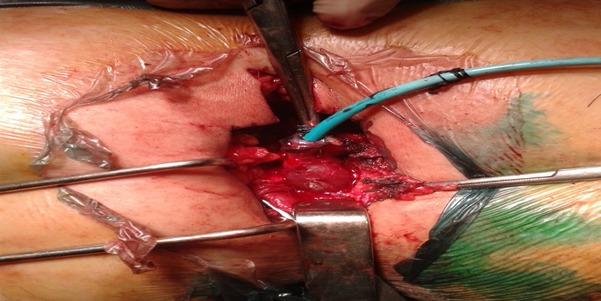
Masse kystique centrée par un drain ( photo en per opératoire)

Patiente âgée de 52 ans diabétique sous glucophage admise pour douleurs pelviennes évoluant depuis 3 mois associés à des ténesmes et constipation chronique, l'examen clinique n'objectivait pas de masse palpable alors que le toucher rectal trouvait un bombement de cul de sac de douglas a droite sans lésions intraluminal. Le bilan biologique était en faveur d'un syndrome inflammatoire biologique avec une CRP à 53 et une VS à 38. La rectoscopie avait noté une compression extrinsèque mais sans tumeur rectale L’échographie (Figure 1) avait montré une image hypoechogene de 4 cm latérovésicale. Le scanner abdominale (Figure 2) et l'IRM (Figure 3, Figure 4) avaient conclu à une tumeur kystique retro rectale. L'intervention chirurgicale a été mené par voie postérieure trans sacré (abord de kraske avec résection du coccyx) (Figure 5), l'exploration avait trouve une formation kystique a paroi propre accolé a la face postérieure du rectum, il a été réalisé une exérèse de la masse kystique a paroi fermé. L'examen anapath avait conclu a un kyste vestigial retro rectal.

## Discussion

Les tumeurs rétro rectales se développent dans l'espace limité en avant par le rectum, en arrière par la pièce sacro-coccygienne, en bas par les releveurs et les muscles anococcygiens, latéralement par les uretères et les vaisseaux iliaques. Fréquentes chez l'enfant et alors souvent malignes, elles sont en général évidentes avec un développement exophytique. À l'inverse, chez l'adulte, ce sont des tumeurs rares, le plus souvent bénignes, asymptomatiques, avec une prédilection féminine [1, 2]. Les kystes et tumeurs d'origine vestigiale y sont les moins rares à côté des chordomes. Chez l'adulte, les kystes vestigiaux sont des tumeurs initialement bénignes qui dégénèrent en carcinomes glandulaires ou épidermoïdes avec une fréquence inférieure à 10% pour les kystes entéroïdes et comprise entre 15 et 25% pour les tératomes [3–5]. L'imagerie par échographie transrectale ou sus pubienne confirme la consistance kystique de la lésion et montre sa situation rétro rectale et sa nature liquidienne. La TDM et l'IRM confirment le siège de la lésion, et permettent un diagnostic de bénignité ou de malignité et précisent l'extension locorégionale [6]. Une rectoscopie et une fistulographie compléteront éventuellement l'exploration en cas de doute diagnostic ou suspicion d'une duplication rectale [6]. La réalisation d'une ponction de la lésion est contre-indiquée car elle est insuffisante pour établir un diagnostic positif. Elle est également associée à un risque d’ infection en cas de méningocèle, de fistule cutanée et de dissémination tumorale en cas de carcinome [1, 6]. La voie d'abord dépend de la localisation du kyste. Les lésions situées en dessous de S3 et sans extension aux viscères pelviens sont abordées par voie périnéale [1, 4, 5]. C'est en règle la position ventrale et la voie de Kraske qui sont utilisées. La résection transanale, dangereuse lorsque le kyste est dégénéré, est réservée aux kystes de moins de 4 cm [1]. Un développement abdominal au-delà de la racine S3 impose un abord abdominal isolé ou combiné, simultané ou différé [1, 5]. La voie coelioscopique est inappropriée pour ce type des lésions. L'exérèse doit etre faite en totalité et en monobloc en raison des risques de récidive lorsqu'elle est incomplète [1]. Pour les tumeurs bénignes, les séquelles après chirurgie sont rares, car la dissection est facilitée par un plan de clivage péri kystique naturel; le risque est surtout celui de la récidive locale survenant dans 10 à 15% des cas. En cas de tumeur dégénérée, les métastases sont fréquentes. Chez l'adulte, leur pronostic est mauvais malgré une chirurgie mutilante, avec des sacrifices osseux et nerveux, éventuellement associée à une radio chimiothérapie, avec une médiane de survie inférieure à deux ans [1, 3, 5].

## Conclusion

Les tumeurs rétro rectales kystiques et solides sont de natures très variées. Les plus répandues sont les chordomes, tumeurs solides à malignité locale importante surtout rencontrées chez l'homme, à égalité de fréquence avec les tumeurs kystiques vestigiales, le plus souvent bénignes, prédominant nettement chez la femme. Ces kystes congénitaux sont souvent asymptomatiques, parfois révélés par des douleurs ou des troubles neurologiques devant faire suspecter une dégénérescence. Leur éventuelle surinfection ne doit pas être confondue avec un abcès fistuleux, de traitement différent.
